# Report on the Origin and Progress of the Cholera at Quarantine, Staten Island, N. Y.

**Published:** 1849-02

**Authors:** 


### ECLECTIC DEPARTMENT,

### AND SPIRIT OF THE MEDICAL PERIODICAL PRESS.

---

*Report on the Origin and Progress of the Cholera at Quarantine, Staten Island, N. Y.* By ALEXANDER B. WHITING, M. D., Health Officer.

QUARANTINE, STATEN ISLAND,
December 11, 1848.

TIMOTHY R. HIBBARD,
Chairman Sanatory Committee of Board of Health.

SIR:—In compliance with a resolution of the Committee, I submit an outline of the origin, progress, and character of the cholera as it has prevailed at Quarantine recently. * * * * * *

On the second of December, the packet ship New York arrived at Quarantine with a number of persons sick, having lost seven during the last week of her voyage, with a disease that has since proved to be Asiatic Cholera. The New York left Havre on the ninth of November, with three hundred and thirty-one steerage passengers, twenty-one cabin, and thirty-three crew; a total of three hundred and eighty-five. All continued well until the twenty-fifth, Saturday, when one of the steerage passengers, a German, aged twenty-nine, in robust health, was attacked with vomiting and purging, accompanied by cramps of the muscles of the upper and lower extremities. The Captain supposed it to be cholera morbus, and prescribed judiciously for the symptoms, but they continued until the third day, when death occurred.

The next case was on the 26th, Sunday, when an old man aged sixty-two, in feeble health, was attacked with vomiting and purging, with coldness of the whole body, and violent cramps and spasms. He died on the second day after the attack. Monday and Tuesday, 27th and 28th, two cases occurred. A girl aged five years, died in two hours, and a boy also, aged five, died in four and a half hours after their first attack, both perfectly well previously. Wednesday, 28, a man aged forty, was attacked at 8 o'clock, A. M, and died at 3 P. M., of the same day. On Thursday two children sickened and died, after six and eight hours' illness.

The ship came to anchor at Quarantine on Friday night, and from that time until Sunday noon, when the passengers were landed, twelve new cases occurred.

The best means of arresting the spread of the disease appeared to me to be to remove them from the confinement of the ship, and to separate the sick from the well. A steamboat was engaged to bring them to the Public Store Docks. The sick were sent to an excellent hospital room, and good nursing and medical attendance immediately provided.

The well have been suitably provided for, and strict attention to diet and cleanliness enforced; their clothing has been thoroughly aired daily, and the whole enclosure subjected to the disinfecting influences of Mr. Grant's valuable Disinfectant. I trust that these means may serve to modify, and soon entirely subdue the disease that appeared to be increasing its malignancy and victims on ship-board.

Since the arrival of the ship at Quarantine, eighteen cases have occurred, making, with the twelve taken from the ship, thirty cases. of which number twenty have died. The whole number from the first case at sea, has been thirty-seven, of which twenty-seven have proved fatal.

The disease was considered by Captain Lines as cholera morbus, and treated by him as such, with calomel and ipecacuanha opiates, warm drinks and mustard, and heat externally. Vomiting and purging, cold clammy skin, cramps and spasms were observed by him in several of the cases, but not the peculiar character of the evacuations.

In the cases that have occurred here, diarrhœa has preceded in only a few, or about one-third. A majority were attacked in the morning, between the hours of two and eight.

Most of them have presented all or most of the following symptoms:—Vomiting and purging of thin discharges, sometimes at first, light brown, but generally from the first, of a white or yellowish white or pearl color, with white flocculi, forming a thicker whitish sediment on standing a short time. They are well described as *rice water* evacuations. In some cases a half gallon has been vomited, but generally in smaller quantities. A child six years old vomited fully this quantity at once, had no other evacuations, and afterwards recovered.

The vomiting is usually accompanied by great uneasiness and pain, particularly at the epigastrium. In some cases vomiting has existed without purging, and vice versa. In several cases neither vomiting nor purging, but the stomach and bowels were found filled after death with the same fluid. One or more large worms, the Lumbrici, have been discharged, either by vomiting or the bowels, in a large majority of cases. This fact has been before remarked.

The tongue and breath are icy cold; sometimes the tongue is clean, but generally slightly coated. The voice becomes weak and husky, and with a great effort the patient speaks in a thick whisper.

The skin assumes a dark livid color, becomes cold and clammy, and when pinched up, remains so for a short time.

The countenance wears a haggard, sunken look, the eye is dull and heavy, although the pupil is somewhat dilated.

The extremities are shrivelled, the fingers and toes, and the nails resembling those that have been long in the water, and of a purple hue.

All the cases have been affected with cramps and spasms of the extremities and abdomen, in some slight, but generally a very painful symptom.

The pulse, from the inception of the real attack, becomes small and frequent, from 110 to 140, according to the progress of the disease, and in the stage of collapse, entirely lost at the wrist for hours.

The breathing labored and hurried, and in the cases where the spasms were severe, occasionally suspended momentarily.

In some of the cases all these symptoms were present, while in others only a few of them existed. The number or *apparent* violence of the symptoms form no criterion for the prognosis. Fatal results followed in a number of cases in a few hours, where the dejections were slight, and spasms and other violent symptoms were absent. Death seems to ensue from the oppression of the vital organs, and the nervous system, by some giant poison. The wretched expression of countenance, the icy tongue and breath, the sunken eye, reveal this most strongly.

In this disease there has been but one stage—that of collapse—although every pains have been taken to detect the first deviations from health, directions given to all to communicate them at once, and persons employed to inspect them constantly, and a physician to pass among them at all hours of the night and day. The first intimations are the extreme symptoms, defying the most prompt and decided remedies.

The appearances after death are those that have been usually observed after death from cholera. A shrunk and shrivelled livid exterior, a gorged and congested condition of the internal organs. The meninges and substance of the brain, and all its vessels, unusually red, either from actual congestion, or the retention of the *cruor* of the blood while the serum is drained off. The lungs, liver, spleen, heart, intestines and kidneys, present the same deepened color. I have observed no alterations of structure or softening.

The bladder is contracted to an extremely small size. I should have observed that there is an entire suppression of urine in most of the cases.

In several cases that I have examined, the entire mucous lining of the bronchial tubes has been much injected.

That this is Asiatic Cholera, cannot be doubted. From the commencement of the disease to the present time, thirty-seven cases have occurred, twenty-seven of which have died in an average period of ten hours. The average duration of the disease in the children that have died, has been four hours.

A new feature has appeared in the history of this disease, in the fact that six persons have been affected in a similar way, who had been but for two days exposed to contact or proximity to these people.

Nothing like cholera existed at this place at the time of the arrival of the ship New-York. When her passengers were removed to the public stores, they were occupied by about seventy persons, who had just recovered from other diseases. One of these, a man just recovering from a fractured patella, assisted in the removal of the patients. This was on Sunday; on the Wednesday following, he was attacked with violent symptoms of cholera, and died the same day. A woman who had been a nurse, without having any communication with these people, but occupying another room in the same building, was attacked, and died the same day, with all the symptoms of cholera. A man who had been discharged and gone to the City of New York on Monday, and had remained a *little over a day* in this same enclosure, was returned from the City as a case of cholera, and died also on Wednesday. On perceiving this communication of the disease to the convalescents, I immediately sent them away and distributed them through the other hospitals, since which, three others have been attacked, two of whom have died, but none other than those at first exposed at the public stores, have been affected. These had all been inmates of the hospital for weeks, were ready to be discharged, and had but a limited exposure for forty-eight hours to the influences of the disease.

At the time the first cases occurred, November 25th, the ship was in N. lat. 42°, long. 61°, about 140 miles S. S. W. from Sable Island. On the 23d and 24th, the two days preceding the appearance of the cholera, the wind was N. N. W. On the 25th it changed to the southward, with squalls and rain. In the morning the barometer was at 30 inches, and fell during the day to 29½ inches; thermometer 60° Fahrenheit. Sunday and Monday, 26th and 27th, wind westerly, and fresh, Tuesday, 28th, moderate from N. W.; barometer 30, thermometer 42°.

I have given these particulars of the wind and weather in connection with the dates of the first appearance of the disease, that all aid may be given to our attempts to account for its origin. Here its actual manifestations commenced. Did it originate in a casual but unusual mingling of certain elements or condition of the elements, or from contact with an advanced wing of the grand cholera army, or from the developement of seeds latent and waiting for peculiar culture? Here we are met by interesting and peculiar facts.

All the persons who have been attacked from the first case on board ship to the last, excepting the inmates of the hospital, have been from among two hundred and seventy Germans who have been living in Havre and its environs, where there has not been a single case of cholera. These persons were originally from Germany, mechanics, and flourishing, until by the triumph of liberty and equality, the native French artisans have succeeded in inducing the public to withdraw their patronage, and the municipal authorities to proscribe them.

The consuls of their native countries have come forward and provided for their emigration to America. The question arises, is there not some difference between these and the other passengers that must enable us to account for the fact.

The only one that may be adduced, is the depressing influence of grief at being driven from their homes and flourishing trades; but this is not apparent in their appearance or manner. They enjoyed promiscuously, with the other passengers, the best accomodations, and I am assured by Capt. Lines, that their fare was the same with the other passengers.

I have examined their provisions on board ship, casks of bacon, rice, flour, beans, biscuit and potatoes, unheaded and exhibited to me, as it has been dealt to them, and I am sure that more wholesome or sweeter provisions could not be provided.

They are all healthy and robust—have not been exposed to the cholera at home, and have since leaving their port of departure, shared, equally with the exempt, the comforts and privations of a sea voyage, variations of wind and weather, have breathed the same air, and fed on the same food.

When I speak of *treatment*, and mention twenty deaths out of thirty cases, I can evidently not be expected to produce a successful plan of treatment.

I will mention the general plan however, as in some cases it has been successful, and in others failed from the fact that the disease presented its first and final stage simultaneously. On admission, the patient is enveloped in warm blankets, next the skin, and warm mustard applied largely to the stomach and bowels and extremities. Hot bricks, bottles of water, or bags of sand applied to various parts of the body, and a stream of hot air conveyed from a hot air apparatus under the bed clothes. With this is combined as much friction with hot tincture of capsicum as can be carried on under the bed clothes without exposure of the body to the air. This is done in all cases, and is an efficient mode of restoring warmth to the surface, if practicable.

Various internal means of treatment have been tried. In a number of cases, Dr. Joseph Brown's practice of administering mustard emetics, has been adopted, but without the blood letting. Only two cases that were treated in the first stage of the disease in this way, were benefitted, reaction occurring after the emetic, followed immediately by a scruple dose of calomel, combined with Dover's powder. Unless reaction is effected by the emetic, the prostration and irritation of stomach produced by it can only serve to enhance the disease.

In eight other cases, the large doses of calomel capsicum and camphor, as administered in the practice of Dr. Cartwright, of Louisiana, and suggested to me by S. M. Fox, Esq., were carefully tried, combined with the rubbing in of hot tincture of capsicum. But the results did not encourage the continuation of the treatment.

Chloroform has been administered in a number of cases, carefully and repeatedly, and at first gave some hope that it would prove a successful remedy, but no other permanent good has resulted from its use but to relieve the spasms and cramps. For this purpose I have used it in all cases moderately, and if not a cure for all the symptoms, it is an invaluable remedy in subduing one of the most painful symptoms of the disease.

Hot wine whey, and mustard whey have been administered, in combination with other means, particularly in the latter stages of extreme prostration.

The *saline mixture* has also received a careful trial in a number of cases from the commencement of the disease, and although hopefully watched for beneficial results, laid aside as worse than inefficient. If administered in a condition of the system when it might be absorbed, advantage may be derived from it, otherwise it can but increase the irritability of the stomach, the thirst and prostration, and aggravate the disease.

Sugar of lead and opium have been administered in large and small doses, but soon abandoned as impotent.

The treatment that has proved of most service has been calomel in scruple doses, combined with opium and camphor, followed at two or three hours intervals, by smaller doses of calomel, until reaction is indicated by some action of the liver. This plan, combined with the faithful application of external heat, &c., I am satisfied has proved of most advantage in the cases that have come under my notice Every case in which the slightest bilious evacuation has been procured, has commenced to recover from that moment, and although of itself, unable to effect the reaction necessary for its own peculiar action, calomel will doubtless always prove the most potent auxiliary in the catalogue of remedies for cholera.

No one specific can ever control a disease whose nature is made up of so many complications, an obstinate exsanguination and paralysis of the surface, with a fearful congestion of all the internal vital organs, and a derangement of the nervous system, indicated by convulsions of every portion of the body.

December 18th. Since the above was submitted, I have had further opportunity to observe the character and treatment of the cholera, existing at the Marine Hospital. Since the 11th December to date, there have occurred thirty-three new cases. All but three of these have been from the same class of Germans from Havre, as the other cases. One, and the only one that has occurred among the French passengers of the New York, was a Frenchman from Paris, a fatal case.

Two others were old inmates of the hospital, of Irish Nativity. They were just recovering from typhus fever, and located in a hospital most distant from the cholera hospital. One was attacked on the 10th, the other on the 11th of December, with all the symptoms of the disease, proving fatal on the third day in the first case. The other is convalescing. No intercourse between these patients and the cholera cases can be detected, neither of the convalescents that were at first returned from the public stores, and were afterwards attacked, had visited their wards, and their physicians in attendance had been but little among the cholera patients.

The whole number of cases, thus far, at Quarantine, has been sixty-three. Of these twenty-nine have died, A large proportion have been children under fourteen years, twenty, or about one third of the whole number having been of this class.

Most of them passed through the first attack of the disease, and died from subsequent congestion or effusion in the brain.

Of the thirty-three cases that have happened since my report of the 11th, I am glad to state that only nine have been fatal. And as there appears to be no difference in the severity of the symptoms at the outset of the disease, I cannot but attribute the diminished fatality to a more happy plan of treatment.

From the results of the first thirty cases, and post mortem revelations, I became convinced that the stimulating plan was not the treatment for *this* cholera, and abandoned at first the mustard, then the capsicum, ammonia, brandy, wine whey, etc., and relied on calomel in large doses, with opium, Dover's powder, and camphor.

With regard to camphor, even though it has been always lauded, and by some as *the specific* in cholera, I entertain suspicions of its utility.

The treatment I have now adopted and adhere to, from its decided agency in controlling the symptoms and inducing early reaction, is the exhibition of moderate doses of calomel, with morphine, at short intervals. Five grains of calomel, with a quarter of a grain of sulph morphia, is at first given to an adult; in a half of an hour, or one hour, a scruple dose of calomel is exhibited, and is usually retained; afterward, a pill of Cal. grs. V, Sulph Morphine gr 1-4, is given each honr, two hours or three hours, as the effect may indicate. This is observed in the subsidence of the pain and spasms, the diminished quantity and frequency of the evacuations, the return of warmth, and the restoration of the pulse.

This treatment is continued until some indications of bilious action appear; the first is usually a change of color and consistence from the light, thin, rice water, to a greenish, and then brown or brownish yellow color. The evacuations from the stomach and bowels will frequently continue green, or of the color of sulphate of copper, for hours, but I have not known a single case to relapse where this effect had once been produced.

I was led to substitute the morphine for opium, from its being less liable to disturb the stomach or to produce narcosis, an effect to be deprecated in this stage of congestion, except it result naturally from the obviation of pain and excitement.

In children, however, under six or seven years, I have used Dover's powder in preference to morphine, as being more manageable in regard to the dose. A very simple remedy, but one that I have used in children with happy effects, has been the tea of the spearmint, given hot in the first stages, and afterwards cold, in a small quantity, a large spoonful occasionally.

The most valuable external means is the stream of hot vapor of alcohol, poured over the patient by a very simple apparatus at the foot of the bed. This is a large alcohol lamp placed under a sheet iron cylinder, with a pipe running from it. The lamp is placed on the floor, and the tube with an elbow, and terminating in a large funnel to elevate the clothes, is inserted under the bed clothes.

This and hot mustard applications are the only external means that I rely on. They are potent, and can be continued without the fatigue or exposure of the patient, a paramount desideratum, as there is plenty of both to contend with as the inevitable effects of the disease.

I should do injustice, did I not mention the faithful attention of the physicians of my staff, particularly Dr. F. L. Harris, Deputy Health Officer, and Dr. John Gallaer, who has devoted his whole time and care faithfully to these patients, through all hours of the night as well as the day.

